# Case Report: Adjusting Seat and Backrest Angle Improves Performance in an Elite Paralympic Rower

**DOI:** 10.3389/fspor.2021.625656

**Published:** 2021-02-11

**Authors:** Anna Cecilia Severin, Jørgen Danielsen, Jørgen Falck Erichsen, Sindre Wold Eikevåg, Martin Steinert, Gertjan Ettema, Julia Kathrin Baumgart

**Affiliations:** ^1^Department of Neuromedicine and Movement Science, Center for Elite Sports Research, Norwegian University of Science and Technology, Trondheim, Norway; ^2^Department of Civil and Environmental Engineering, Center for Sports Facilities and Technology, Norwegian University of Science and Technology, Trondheim, Norway; ^3^Department of Mechanical and Industrial Engineering, Norwegian University of Science and Technology, Trondheim, Norway

**Keywords:** kinematics, paraplegia, elite athlete, equipment modification, rowing ergometer

## Abstract

Paralympic rowers with functional impairments of the legs and trunk rely on appropriate seat configurations for performance. We compared performance, physiology, and biomechanics of an elite Paralympic rower competing in the PR1 class during ergometer rowing in a seat with three different seat and backrest inclination configurations. Unlike able-bodied rowers, PR1 rowers are required to use a seat with a backrest. For this study, we examined the following seat/backrest configurations: conA: 7.5°/25°, conB: 0°/25°, and conC: 0°/5° (usually used by the participant). All data was collected on a single day, i.e., in each configuration, one 4-min submaximal (100 W) and one maximal (all-out) stage was performed. The rowing ergometer provided the average power and (virtual) distance of each stage, while motion capture provided kinematic data, a load cell measured the force exerted on the ergometer chain, and an ergospirometer measured oxygen uptake (${\dot{\text{V}}\text{O}}_{2}$). Where appropriate, a Friedman's test with *post-hoc* comparisons performed with Wilcoxon signed-ranked tests identified differences between the configurations. Despite similar distances covered during the submaximal intensity (conA: 793, conB: 793, conC: 787 m), the peak force was lower in conC (conA: 509, conB: 458, conC: 312 N) while the stroke rate (conA: 27 conB: 31, conC: 49 strokes·min^−1^) and ${\dot{\text{V}}\text{O}}_{2}$ (conA: 34.4, conB: 35.4, conC: 39.6 mL·kg^−1^·min^−1^) were higher. During the maximal stage, the virtual distances were 7–9% longer in conA and conB, with higher peak forces (conA: 934 m, 408 N, conB: 918 m, 418 N, conC: 856 m, 331 N), and lower stroke rates (conA: 51, conB: 54, conC: 56 strokes·min^−1^), though there was no difference in ${\dot{\text{V}}\text{O}}_{2\text{peak}}$ (~47 ml^−1^·kg^−1^·min^−1^). At both intensities, trunk range of motion was significantly larger in configurations conA and conB. Although fatigue may have accumulated during the test day, this study showed that a more inclined seat and backrest during ergometer rowing improved the performance of a successful Paralympic PR1 rower. The considerable increase in ergometer rowing performance in one of the top Paralympic rowers in the world is astonishing and highlights the importance of designing equipment that can be adjusted to match the individual needs of Paralympic athletes.

## Introduction

Paralympic rowers compete in three classes; PR1 for athletes with no leg function, minimal/no trunk function, and poor sitting stability, PR2 for athletes with limited/no leg function and functional use of the trunk, and PR3 for athletes with residual leg function (https://bit.ly/370Scrz, accessed December 4, 2020). While Paralympic rowers compete over the same 2000-m distance as Olympic rowers, the current world records for male and female PR1 rowers are around 3 min slower (~7 vs. 10 min) than the world record for able-bodied rowers (www.worldrowing.com/events/statistics, accessed October 22, 2020). The faster times in able-bodied rowers are mainly because of the ability to utilize their whole body during the rowing task (Baudouin and Hawkins, 2002; Maestu et al., 2005; Van Soest and Hofmijster, 2009). In addition, while both PR1 and PR2 rowers use a fixed seat, PR1 rowers have less sitting stability than PR2 rowers, and are thus required to be strapped into their seat during competition. Therefore, PR1 rowers rely predominantly on their arms and shoulders to generate the boat speed (Cutler et al., 2017).

Regardless of whether rowers are able to actively utilize their legs or not, the purpose of the sport is to cover the race distance as fast as possible. The boat speed is dependent on the propulsive force produced (Baudouin and Hawkins, 2004), which in turn depends on the physical capabilities and technique of the rower, and the configuration and design of the equipment (Baudouin and Hawkins, 2002; McGregor et al., 2004). Burkett (2010) highlighted that the seat can be modified to match the individual needs of the athlete in Paralympic rowing, and the World Rowing Federation (WRF) currently has few restrictions with regard to seat configurations. The only regulations state that that PR1 athletes must have a backrest on their seat and use a trunk strap for safety purposes with specifications on how these straps should be formed and function (Rolland and Smith, 2017). While using and adapting equipment to match the requirements of the individual Paralympic rower may have a large effect on performance, such effects have not been reported in the literature.

To date, most research on seat modifications for Paralympic performance has been conducted on wheelchair sports (e.g., Costa et al., 2009; Vanlandewijck et al., 2011; Van Der Slikke et al., 2018). Vanlandewijck et al. (2011) found that utilizing a more posteriorly inclined seat can benefit seating stability but highlighted that it may also have negative effects on performance. This was because the increased hip flexion angle and pelvic posterior tilt appeared to reduce the trunk and shoulder range of motion (ROM). Contrary to wheelchair propulsion, rowing propulsion is comprised of a backward pull and thus relies more on trunk extension. It is therefore possible that adjusting the inclination of the backrest, and thereby allow more trunk extension, may compensate for an inclined seat. This may, in turn, allow the athlete to regain some of the restricted motion and improve performance. However, it remains unknown if this applies to Paralympic rowers with minimal trunk function. This case report therefore aimed at assessing the effects of a more inclined seat and backrest on rowing performance in a multiple Paralympic PR1 world champion.

## Case Description

The participant was an elite female Paralympic PR1 rower (age: 30 years, height: 1.80 m, body mass: 60 kg), who acquired an incomplete spinal cord injury in 2008 at the level of the 10th thoracic vertebra, leaving her with minimal trunk function and reduced sitting stability (see https://bit.ly/370Scrz for a description of the tests performed during classification). At the time of the data collection she did not have any additional injuries, was in good health, and trained ~28 h per week. Written informed consent was obtained prior to data collection, and the testing complied with the declaration of Helsinki.

## Laboratory Testing and Measured Variables

**Figure 1:**
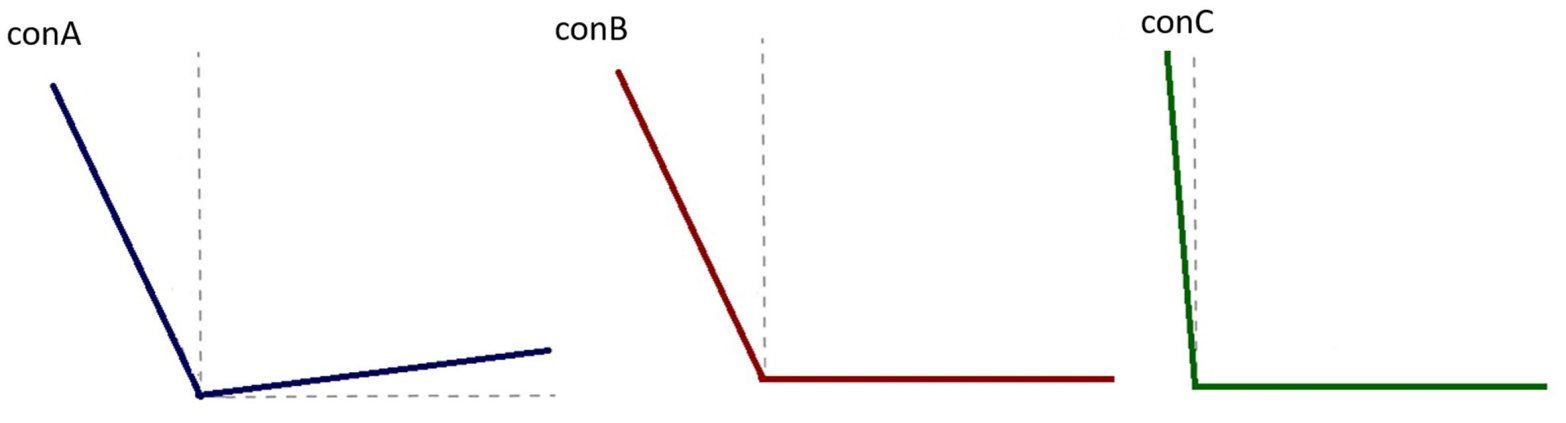
Illustration of the three seat configurations used during this study. conA had a 25° incline of the backrest from the vertical plane, with a 7.5° incline of the seat to the horizonal plane. conB had the same backrest incline as conA (25° to the vertical) and a flat seat (0° to the horizontal). conC, which was the participant's usual set up, had the backrest inclined to 5° from the vertical plane and a flat seat (0° from the horizontal plane).

The participant attended the laboratory on 2 consecutive days, with pilot testing and familiarization on day 1, and the data collection on day 2. A custom-made test seat replaced the original seat on a Concept2 rowing ergometer (Concept2, Morrisville. VT. USA). The seat and backrest inclinations were adjustable but the seat itself was stationary (non-sliding). Based on the pilot testing from day 1, the three seat configurations analyzed on day 2 were: seat 7.5° (from horizontal) and backrest 25° (from vertical) (conA), seat 0° and backrest 25° (conB), and her usual configuration seat 0° and backrest 5° (conC) (Figure 1). The participant was strapped into the seat with one strap across her upper thighs, and one strap around her lower trunk, similar to her competition set-up.

The Concept2 software provided the virtual rowing distance covered (henceforth referred to as distance) and the average power output, while a Futek Miniature Load Cell (Futek LCM200; capacity, 250 lbs.; nonlinearity 0.5%; hysteresis 0.5%; weight 17 g; Futek Inc., Irvine, CA) was used to record the instantaneous force exerted by the participant on the chain of the ergometer (200 Hz). The load cell was calibrated against a range of forces of known magnitude employing calibrated weights (linear correlation r^2^ = 0.999). Kinematics were collected by a 10-camera system (Oqus, Qualisys AB, Gothenburg, Sweden) recording at 100 Hz. Bilateral symmetry was assumed, and retroreflective markers were attached to the participants left side on the 2nd toe, lateral malleolus, lateral femoral epicondyle, greater trochanter, iliac crest, the spinous processes of the T10 and C7 vertebrae, acromion process, lateral epicondyle of the humerus, and styloid process of the radius. One additional marker was placed on the ergometer handle and one on the flywheel, allowing for identifying strokes. Rate of oxygen uptake (${\dot{\text{V}}\text{O}}_{2}$) was recorded using an ergospirometer with a mixing chamber (Oxycon Pro, Jaeger GmbH, Hoechberg, Germany) and a mouthpiece (Hans Rudolph Inc, Kansas City, MO, USA). Prior to testing, the gas analyzer was calibrated against a known mixture of gases (15% O_2_ and 5% CO_2_) and ambient air. Calibration of the flow transducer was manually performed with a 3L high precision syringe (Hans Rudoph Inc., Kansas City, MO, USA). Heart rate (HR) was monitored using an H10 Polar heart rate monitor (Polar Electro Inc., Kempele, Finland). Blood lactate concentration (BLa) was assessed with the Lactate pro 2 (Arkray Inc., Kyoto, Japan). Subjective rate of perceived exertion (RPE) was measured on a 6–20 Borg scale (Borg, 1982).

The data collection protocol consisted of three 4-min stages performed at 80 W, 100 W, and an all-out effort in each of the three seat configurations. During the all-out stage, the participant was instructed to row as hard as she could for the 4 mins and pace herself so that she reached exhaustion toward the end of the stage. Maximal exhaustion was considered reached if 2 of the 3 following criteria were met: (1) the self-reported max heart rate from the participant, (2) respiratory exchange ratio over 1.15, and (3) an RPE of 18 or higher. The participant was allowed 2–3 mins rest between stages and 30 mins between the different configurations. The 80 W stages were considered familiarization stages and were not included in the analysis. The 100 W (SUBMAX) stages provided steady-state responses while the all-out (MAX) stages provided peak responses. Performance (i.e., distance covered during MAX), biomechanical, and physiological data were recorded throughout the 4-min stages. RPE was recorded after each stage and a BLa was measured from the earlobe directly after SUBMAX, and 1 and 3 min after MAX.

**Figure 2:**
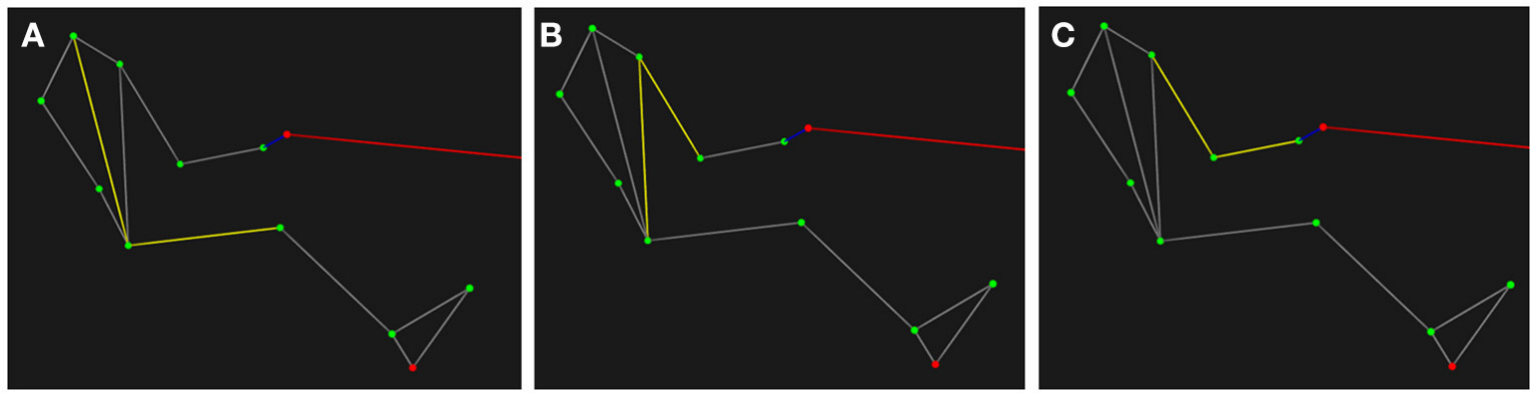
Definition of the angles calculated from the kinematic data (indicated by the yellow lines). **(A)** Trunk: angle created between the thigh and neck using markers on the lateral femoral epicondyle, the greater trochanter, and the spinous process of C7. **(B)** Shoulder: angle created between the greater trochanter, acromion process, and lateral humeral epicondyle. **(C)** Elbow: angle created between the acromion process, lateral humeral epicondyle, and radial styloid process.

SUBMAX steady-state ${\dot{\text{V}}\text{O}}_{2}$ and HR data were calculated by averaging the final 60 s of each stage. For MAX, the data was analyzed using a 30 s moving average for the ${\dot{\text{V}}\text{O}}_{2}$ and 30 s for the HR, and the peak value was identified as ${\dot{\text{V}}\text{O}}_{2\text{peak}}$ and HR_peak_, respectively. Kinematic and force data were analyzed using custom MATLAB code (MATLAB 2019b, Matworks Inc., Nantick, MA, USA). Marker and force data were low-pass filtered using a 4th order Butterworth filter with cut-offs of 7 and 50 Hz, respectively. Elbow and shoulder joint and trunk angles were calculated from marker positions (Figure 2).

The start of each rowing stroke was defined as the point where the handle marker was closest to the flywheel marker. The stroke was divided into a drive and a recovery phase (Cutler et al., 2017), with the end of drive identified as when the handle was farthest away from the flywheel. Eighteen strokes in the middle of each stage were extracted for analysis. For each stroke, timeseries data for the joint angles (trunk, shoulder, and elbow) and force data from the load cell were time-normalized to 101 data points (0–100% of each stroke). In addition, the following discrete biomechanical variables were extracted for the 18 cycles: maximal and minimal joint angles, peak force, impulse (the integral of force over time), the drive phase duration (expressed as % of stroke), the stroke rate, and stroke length (i.e., distance the handle moved during the drive phase).

The joint angles, peak force, impulse, drive phase duration, stroke rate, and stroke length were analyzed in SPSS version 26 (IBM Inc., Armonk, NY, USA). All variables violated the assumptions of a repeated-measures one-way ANOVA so differences between configurations were determined using a Friedmans test with a subsequent Wilcoxon singed-ranks tests for *post-hoc* comparisons. Statistical significance of all *post-hoc* tests was accepted at an alpha level of 0.017 (0.05/3 ≈ 0.017 following Bonferroni adjustments). Cohen's D was used to indicate effect size, and was considered small if *d* < 0.5, moderate if 0.5 < *d* < 0.8, and large if *d* > 0.8 (Cohen, 1988). The drive phase of the normalized time series were analyzed using Statistical Parametric Mapping (SPM) (github.com/0todd0000/spm1dmatlab, accessed March 17, 2020) by employing paired samples *T-*tests (Pataky et al., 2016). The time interval used for the SPM analysis was chosen as the drive phase duration for conA and conB that occurred last (38% for SUBMAX from conA, and 51% for MAX from conB). Statistical significance for the SPM analyses was accepted at an alpha level of 0.05.

## Outcomes and Results

**Figure 3:**
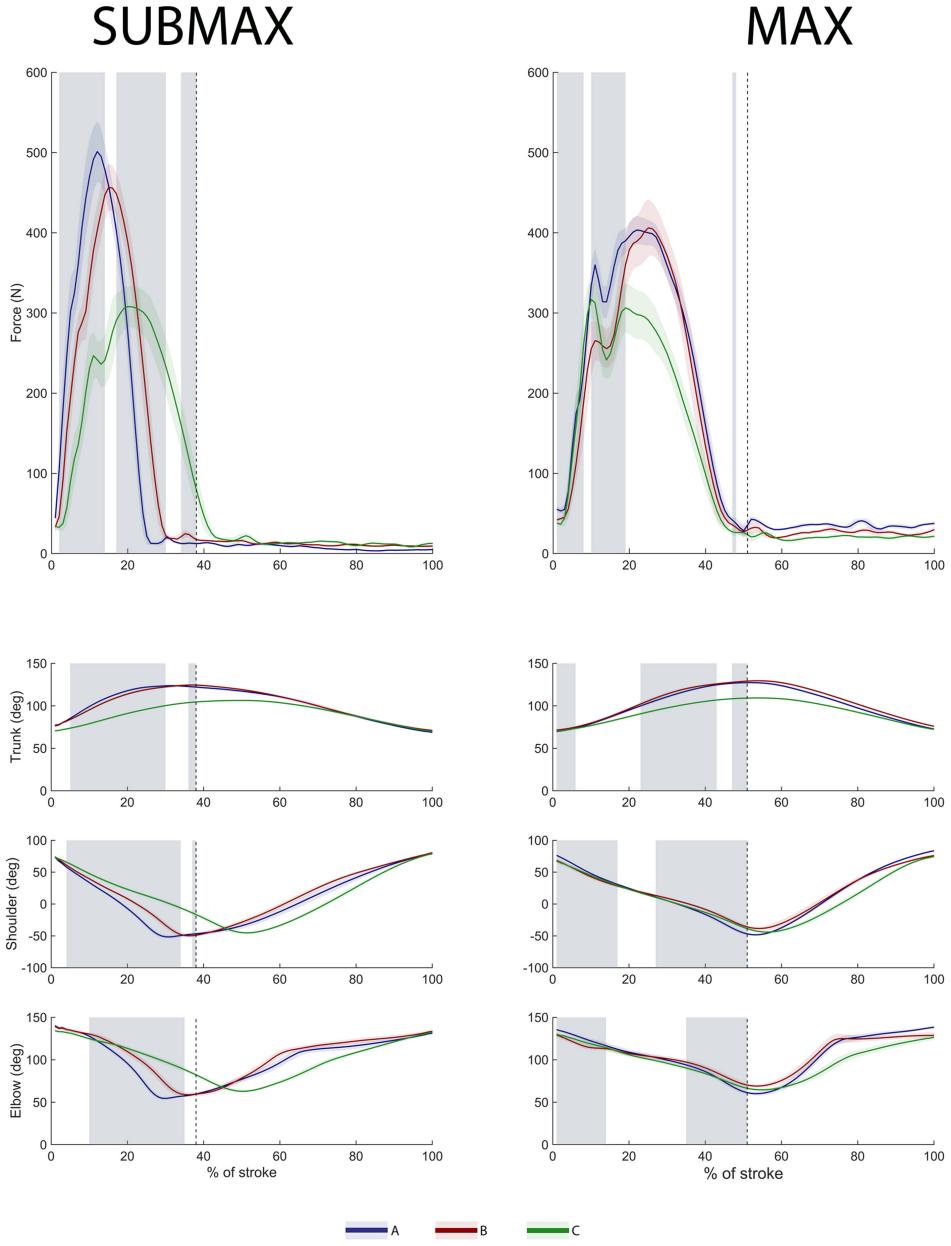
Mean ± standard deviation of the handle force and joint angles for the 3 seat configurations (conA, conB, and conC) for 0–100% of a stroke. The vertical dashed line indicates the point of phase shift (between drive and recovery phase), up until which the SPM analysis was performed. The shaded area in gray indicates when there was a significant difference during the drive phase between conA and conB since conC was statistically different to the other configurations during most of the drive phase.

The Friedmans tests indicated significant main effects of seat configurations on all tested variables (χ^2^(2) < 36.000, *p* < 0.05). Table 1 shows the results of the *post-hoc* comparisons for the biomechanical variables along with descriptive data for the performance and physiological variables. During SUBMAX, conC had significantly lower peak force and impulse coupled with higher ${\dot{\text{V}}\text{O}}_{2}$ and significantly higher stroke rate than conA and conB. Further, although the distance, ${\dot{\text{V}}\text{O}}_{2}$, and RPE were similar between conA and conB, conB had higher HR and significantly lower peak force and impulse. During MAX, longer distances were covered in conA (+78 meters) and conB (+60 meters), compared to conC (Table 1, Figure 3). Peak force was significantly higher and stroke rate was significantly lower in conA and conB compared to conC also at MAX, although these differences were smaller than at SUBMAX.

**Table 1:** Performance, physiological, and biomechanical variables presented as single values or mean ± SD for the three seat configurations tested on day two with statistical comparisons for the three configurations.

	**100 W**						**MAX**					
	**conA**	**conB**	**conC**	**conA v conB**	**conA v conC**	**conB v conC**	**conA**	**conB**	**conC**	**conA v conB**	**conA v conC**	**conB v conC**
Distance (m)	793	793	787	–	–	–	934	918	856	–	–	–
Power output (W)	101	101	99	–	–	–	165	157	127	–	–	–
${\dot{\text{V}}\text{O}}_{2}$ (mL·kg^−1^·min^−1^)	34.4	35.4	39.6	–	–	–	46.3	46.2	47.4	–	–	–
HR (bpm)	157	166	176	–	–	–	188	188	187	–	–	–
BLa (mmol·L^−1^)	3.6	6.4	11.3	–	–	–	21.8	23.5	18.4	–	–	–
RPE	11	12	14	–	–	–	20	19	19	–	–	–
Peak Force (N)	509 ± 40	458 ± 30	312 ± 28	0.003^α^	<0.001^α^	<0.001^α^	408 ± 17	418 ± 29	331 ± 23	0.170	<0.001^α^	<0.001^α^
Impulse (N·s)	172.2 ± 8.1	157.9 ± 8.7	97.0 ± 8.9	<0.001^α^	<0.001^α^	<0.001^α^	153.5 ± 5.9	129.9 ± 10.0	102.9 ± 6.5	<0.001^α^	<0.001^α^	<0.001^α^
Drive phase duration (%)	32 ± 1	37 ± 1	51 ± 1	<0.001^α^	<0.001^α^	<0.001^α^	52 ± 1	54 ± 1	56 ± 1	0.001^α^	<0.001^α^	0.001^α^
Stroke rate (spm)	26.5 ± 0.7	30.8 ± 0.8	48.6 ± 1.4	<0.001^α^	<0.001^α^	<0.001^α^	50.9 ± 0.8	54.1 ± 1.2	56.0 ± 0.7	<0.001^α^	<0.001^α^	0.001^α^
Stroke length (cm)	80.5 ± 2	77.7 ± 1.2	67.2 ± 1.3	0.002^α^	<0.001^α^	<0.001^α^	78.7 ± 1.2	77.3 ± 1.7	68.4 ± 1.1	0.006^α^	<0.001^α^	<0.001^α^
Trunk flexion (°)	68.7 ± 0.8	70.9 ± 0.9	69.3 ± 1.2	<0.001^α^	0.170^β^	<0.001^α^	69.8 ± 0.8	71.6 ± 1.1	70.2 ± 1.1	<0.001^α^	0.053	0.004^α^
Trunk extension (°)	123.9 ± 1.5	124.5 ± 1.1	106.4 ± 0.7	0.102	<0.001^α^	<0.001^α^	127.4 ± 1.1	129.6 ± 0.8	109.4 ± 0.9	<0.001^α^	<0.001^α^	<0.001^α^
Trunk ROM (°)	55.2 ± 1.8	53.6 ±1.2	37.2 ± 1.1	0.011^α^	<0.001^α^	<0.001^α^	57.7 ± 1.3	58.0 ± 1.5	39.2 ±1.3	0.446	<0.001^α^	<0.001^α^
Shoulder flexion (°)	82.4 ± 1.4	81.7 ± 1.5	80.1 ± 2.0	0.148	0.004^α^	0.025^α^	84.7 ± 1.4	76.7 ± 3.5	75.1 ± 1.8	<0.001^α^	<0.001^α^	0.094^β^
Shoulder extension (°)	51.8 ± 2.7	50.3 ± 2.2	45.5 ± 2.1	0.064^β^	<0.001^α^	<0.001^α^	48.5 ± 1.6	38.9 ± 3.2	44.2 ± 2.4	<0.001^α^	0.002^α^	0.001^α^
Shoulder ROM (°)	134.1 ± 3.5	132.0 ± 2.6	125.6 ± 3.2	0.043^β^	<0.001^α^	<0.001^α^	133.1 ± 1.7	115.7 ± 4.5	119.3 ± 3.2	<0.001^α^	<0.001^α^	0.018^α^
Elbow flexion (°)	140.1 ± 2.2	139.5 ± 1.8	134.8 ± 1.7	0.446	<0.001^α^	<0.001^α^	139.0 ± 1.7	130.9 ± 3.0	130.4 ± 1.8	<0.001^α^	<0.001^α^	0.586
Elbow extension (°)	54.4 ± 1.8	58.6 ± 3.0	62.5 ± 2.0	0.002^α^	<0.001^α^	0.002^α^	59.9 ± 2.3	68.6 ± 2.0	64.4 ± 2.1	<0.001^α^	0.001^α^	<0.001^α^
Elbow ROM (°)	85.7 ± 3.1	80.9 ± 3.5	72.5 ± 2.6	0.010^α^	<0.001^α^	<0.001^α^	79.1 ± 2.3	62.5 ± 4.3	65.7 ± 2.3	<0.001^α^	<0.001^α^	0.013^α^

*bpm, beats per min; cm, centimeters; d, Cohen's D; m, meters; N, Newtons; p, p-value; spm, strokes per min; $\dot{\text{V}}\text{O}2$, Oxygen uptake; W, watt*.

*Drive phase duration indicates the timing of when the participant transitioned from the drive phase to the recovery phase*.

α *denotes a large effect size (d > 0.8 or d < −0.8)*,

β *denotes a moderate effect size (0.5 < d < 0.8 or −0.8 < d < −0.5)*.

Trunk extension was significantly less in conC compared to conA and conB during both intensities (Table 1, Figure 3). The SPM analysis showed significant differences between configurations conA and conB (shaded areas in Figure 3) in both their force profiles and joint kinematics throughout a large part of the drive phase during both intensities. While the elbow and shoulder joints showed a similar pattern during all three configurations, the timing of their peak flexion significantly differed between configurations during SUBMAX (Figure 3). Though the differences in peak shoulder and elbow flexion and extension angles between conC and the other configurations were small (less than 6.0°), the consistent movement pattern of the participant resulted in these differences reaching statistical significance (Table 1).

## Discussion

In this case report, the performance of an elite Paralympic rower improved substantially during an all-out maximal effort on a rowing ergometer when using adjusted seat configurations. Compared to her usual setup (conC), the configurations with an increased back angle (conA and conB) showed 7–9% improved performance (virtual distance covered) along with significantly higher peak force production, larger impulse, increased trunk motion and longer stroke length coupled with lower stroke frequency.

During SUBMAX, the participant was able to maintain the target power (100 W) in all three configurations, and therefore covered similar distances. However, she had to employ a higher stroke rate in her usual setup (49 strokes·min^−1^) compared to conA and conB, which is considerably higher than what usually is reported for able-bodied rowers (20–36 strokes·min^−1^) (McGregor et al., 2004; Hofmijster et al., 2007). This was to compensate for the lower peak force, lower impulse, and shorter stroke length. The higher stroke rate was achieved predominantly through a shorter recovery phase in conC (conA: 1.53 s, conB: 1.23 s, and conC: 0.60 s), which required an active contribution from the participant to return the handle toward the flywheel before the next stroke. In addition, with a high stroke rate, the participant moved faster and changed the direction of movement more frequently, which required her to continuously overcome larger linear momentum. Further, while the drive phase durations only differed by < 0.1 s between all three configurations, the stroke lengths in conA and conB were ~10 cm longer than conC during SUBMAX. It has been shown in able-bodied rowers, that the amount of positive work done per stroke during rowing is mainly dependent on stroke length (Hofmijster et al., 2007). Consequently, the high stroke rate with a shorter recovery phase, and shorter stroke lengths, would be disadvantageous for producing work. A high stroke rate has further been linked to increased respiratory demands (Saltin et al., 1998; Lindinger and Holmberg, 2011), which is supported by the current study in that the higher ${\dot{\text{V}}\text{O}}_{2}$ in conC indicates a lower efficiency than in conA and conB.

Surprisingly, both peak force and impulse were higher at SUBMAX (work rate 100 W) than at MAX for both conA and conB (work rate 165 and 157 W, respectively). In addition, her stroke rate increased considerably for conA and conB (+83 and +57%, respectively) at MAX compared to SUBMAX, while conC only increased with 15%. It is also noteworthy that the stoke rate only differed by ~10% between the three configurations at MAX, which suggests that she has a “default” stroke rate during “all-out” effort bouts. It seems the participant adopted this “default” stroke rate when performing an “all-out” effort during the testing, which subsequently shortened the time per cycle, and caused the lower impulse and peak force at MAX. Importantly, if the participant adopted this “default” stroke rate, it suggests the “all-out” instruction triggered a rowing technique that was different from the one used when instructed to maintain a target power (i.e., SUBMAX). So, while the participant covered a longer distance at MAX in conA and conB, this was done with a less powerful drive phase. Our findings therefore suggest that she may be able to perform even better if she can maintain a rowing technique at MAX, with a more powerful drive phase and lower stroke rate.

Further, while stroke rate and drive phase duration differed considerably between conC and the other configurations at SUBMAX, they were more similar at MAX, suggesting that the participants “all-out” effort strategy was similar regardless of the seat configuration. Conversely, the gains the participant achieved in peak force/impulse when transitioning from SUBMAX to MAX were noticeably smaller in conC than conA and conB. This was perhaps since she was not able to increase the already high stroke rate much further in conC (+15%), which also resulted in a shorter distance covered. In line with the lower efficiency during SUBMAX, the performance was poorer in conC during MAX, despite similar levels of volitional exhaustion (${\dot{\text{V}}\text{O}}_{2\text{peak}}$, HR_peak_ and RPE) in all configurations.

Even in a participant with minimal residual trunk function, the increased performance in conA and conB was likely associated with the increased trunk motions due to the inclined backrest (Table 1). This supports previous research that linked increased power production to increased trunk ROM in able-bodied male rowers (McGregor et al., 2004). The increased trunk motion likely also triggered the arm movements earlier in the stroke during SUBMAX (Figure 3), which subsequently allowed the more rapid force development and the longer recovery phases (Table 1). The backrest inclination was the same for conA and conB (25°), so the marginally better performance in conA may in part be due to the increased seat inclination (conA: 7.5°, conB: 0°). Speculatively, the inclined seat may have prevented the participant from sliding forward during the strokes and thereby increased her stability. In wheelchair athletes, an inclined seat has been cautioned to have negative effects on performance since it creates “closed” posture with reduced trunk ROM (Vanlandewijck et al., 2011). However, the difference in seat angle between conA and conB caused only minimal differences in trunk angles (flexion: 2.2° and 1.8°, extension: 0.6° and 2.2° in SUBMAX and MAX, respectively), and the more closed posture in conA did not have a negative effect on her performance. Furthermore, wheelchair propulsion and rowing are opposite movements, and it is therefore likely that rowing performance would be more affected by the range of trunk extension and is not as affected by limited trunk flexion as wheelchair performance. This further highlights the importance of allowing trunk extension even for Paralympic rowers with minimal residual trunk function. Overall, this data shows the importance of designing individualized equipment to match the very heterogenous physical capabilities of Paralympic athletes.

## Subject Perspective

Following this experiment, the athlete chose to employ conA and conB during training and has, after a few months, settled with conA. During the experiment, the athlete commented that conA and conB felt “easier and more effective,” and that she “didn't have to use so much energy.” The coach also observed that athlete seemed more relaxed in these adjusted configurations and particularly noticed the lower stroke rate.

## Limitations

A limitation of this case report is that because of time restriction, all configurations were tested on 1 day, with conA first, then conB, and conC last. Despite measures to prevent fatigue, it is possible that the results may in part be attributed to accumulating fatigue. However, the differences between conC and the other configurations are so large that it seems unlikely that these effects would disappear completely even if fatigue was avoided. Some of the significant differences in kinematics between the configurations were also very small (e.g., trunk angle, Figure 3). This was caused by the very consistent movement patterns from the single, experienced rower in this case report, resulting in small standard deviations. However, in elite sport, even such small changes may still affect the athlete's chances of winning a medal or finishing off the podium. Finally, even though differences exist between indoor ergometers and on-water rowing (Shaharudin et al., 2014), ergometers are frequently used by high performance rowers during testing and training (Bjerkefors et al., 2007; Van Soest and Hofmijster, 2009; Cutler et al., 2017). On the ergometer, we saw a 7–9% performance improvement in conA and conB, compared to the participants usual set up. Although non-standardized on-water pilot testing has indicated performance improvements with the adjusted seat, the extent of these during competitions remains to be investigated.

## Conclusion

This case study showed that adjustments to the seat and backrest improved performance by 7–9% in an elite Paralympic PR1 rower compared to her usual configuration during land-based ergometer rowing. The two configurations with increased backrest inclination allowed longer virtual distances, higher peak forces, larger impulses, increased trunk motions, longer stroke lengths, and lower stroke rates compared to the participants usual set-up. It should be acknowledged that the design of the study, where the participant performed three “all-out” tests on a single day, may have resulted in accumulating fatigue and thus affected the results. However, the differences between her usual set up and the adjusted configurations were so large that is seems unlikely that they would disappear completely if fatigue was avoided.

## Data Availability Statement

The data that support the findings of this study are available on request from the corresponding author, AS. The data are not publicly available due to their containing information that could compromise the privacy of the research participant.

## Ethics Statement

The studies involving human participants were reviewed and approved by Norwegian Centre for Research Data (ID 689366). The patients/participants provided their written informed consent to participate in this study. Written informed consent was obtained from the individual(s) for the publication of any potentially identifiable images or data included in this article.

## Author Contributions

AS participated in the conceptualizing of the study, the data processing, statistical analyses, and writing of the manuscript. JF, SW, and MS designed the seat and modified the Concept2 used in the study, participated in the conceptualization of the study, the data collection, and the writing of the manuscript. JD participated in the conceptualization of the study, the data collection, and the writing of the manuscript. GE contributed to the design of the study, the data analysis, and writing of the manuscript. JB participated in the conceptualization of the study, the data collection, statistical analysis, and the writing of the manuscript. All authors have read and approved the final version of the manuscript and agree with the order of presentation of the authors.

## Conflict of Interest

The authors declare that the research was conducted in the absence of any commercial or financial relationships that could be construed as a potential conflict of interest.
